# Call to Enhance Pediatricians' Capacity in Delivering Child and Adolescent Mental Health Services in China

**DOI:** 10.1111/appy.70007

**Published:** 2025-09-07

**Authors:** Meirong Pan, Ni Tang, Jianguang Qi, Zhengjie Zhu, Qingjiu Cao, Tianmei Si

**Affiliations:** ^1^ Peking University Sixth Hospital, Peking University Institute of Mental Health, NHC Key Laboratory of Mental Health (Peking University), National Clinical Research Center for Mental Disorders (Peking University Sixth Hospital) Beijing China; ^2^ Children's Medical Center of Peking University First Hospital Beijing China

**Abbreviations**
CAMHS: child and adolescent mental health services
CAP: child and adolescent psychiatry

1

Mental disorders are leading causes of disability in children and adolescents in China, affecting 30.8 million individuals (Dong et al. 2025). Despite escalating needs, China's child and adolescent mental health services (CAMHS) face severe workforce shortages, exacerbated by systemic inefficiencies with unmet lower‐tier services demands (Jiang et al. 2024). Strengthening pediatricians' CAMHS capacity is a viable solution aligned with WHO's stepped‐care model (WHO Team 2021). Despite global consensus on essential mental health competencies among pediatricians (Foy et al. 2019), significant gaps persist in China, both in targeted surveys and policy frameworks, specifically addressing child and adolescent psychiatry (CAP) competency development among pediatricians.

Our national survey of 537 pediatricians highlights urgency: 496 (92.36%) encountered pediatric mental health cases. Of these, 75.60% reported moderate‐to‐high stress when delivering CAMHS, primarily due to insufficient CAP skills. Only 32.77% received CAP‐specific training, primarily through self‐directed learning (*n* = 84, 47.73%) and conferences (*n* = 82, 46.59%). Strong consensus existed for CAMHS competency development (*n* = 517, 96.27%), prioritizing communication skills, early recognition of mental health conditions, and multidisciplinary teamwork (Figure 1).

**FIGURE 1:**
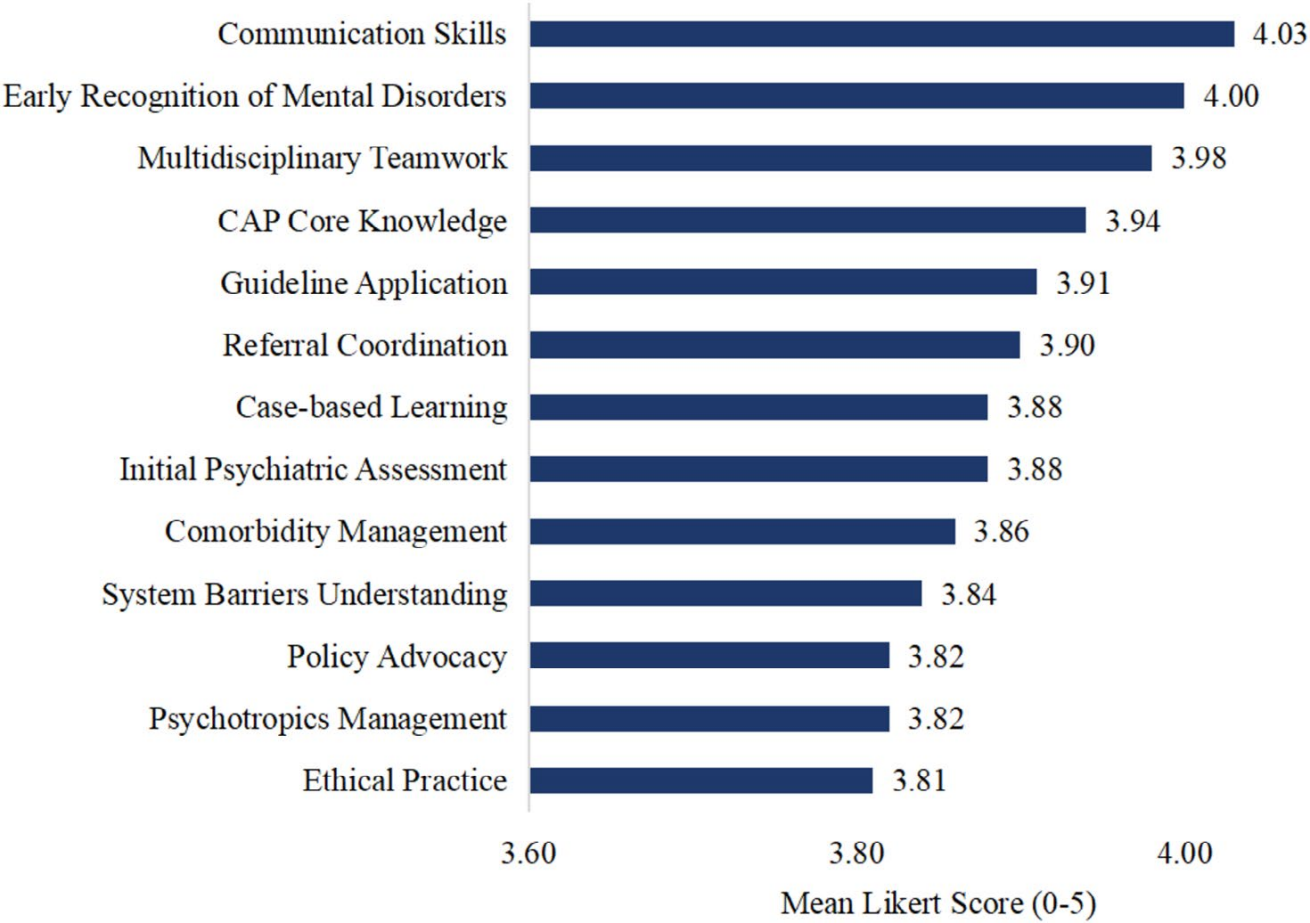
Priority CAP training areas (mean importance rating ± SD, *N* = 517). CAP, child and adolescent psychiatry.

To address the gaps, we urgently call for immediate actions, including: (1) mandatory CAP training adapting best practices to local needs focusing on communication skills and early disorder recognition to boost pediatricians' willingness and competence in CAMHS; (2) a national CAP e‐library, grounded on foundational resources like mhGAP (World Health Organization 2017), to provide standardized content, reduce geographic disparities, and integrate guidelines, lectures, case studies, and regional resources for referrals; and (3) support from the National Health Commission and academic leadership, including policy directives, increased CAMHS funding, and CAP rotation quality assessment.

Enhancing pediatricians' CAMHS capacity through these steps will help China build a sustainable CAMHS workforce for youth needs, improve outcomes, and offer a scalable model for other Asia‐Pacific nations facing similar workforce shortages and seeking to expand CAMHS within primary care frameworks.

## Author Contributions

Meirong Pan conceived the study design and performed data collection, extraction, and analysis, and drafted the paper. Ni Tang conceived the study design and performed data collection, extraction, and analysis. Jianguang Qi conceived the study design and performed data extraction. Zhengjie Zhu performed data extraction. Tianmei Si conceived the study design and performed data collection, extraction, and analysis; drafted and reviewed the paper. All authors reviewed the final manuscript.

## Disclosure

The authors have nothing to report.

## Ethics Statement

This study has been approved by the Ethics and Clinical Research Committees of Peking University Sixth Hospital [(2024) Ethics review number (68)] and was performed in accordance with the Declaration of Helsinki with the Medical Research Involving Human Subjects Act (WMO).

## Conflicts of Interest

The authors declare no conflicts of interest.

## Data Availability

Data sharing not applicable to this article as no datasets were generated or analyzed during the current study.
